# How Does Spatial Injustice Affect Residents’ Policy Acceptance of the Economic–Social–Ecological Objectives of Construction Land Reduction?

**DOI:** 10.3390/ijerph20042847

**Published:** 2023-02-06

**Authors:** Keqiang Wang, Jianglin Lu, Hongmei Liu

**Affiliations:** 1 School of Public Economics and Administration, Shanghai University of Finance and Economics, Shanghai 200433, China; 2 Technology Innovation Center for Land Spatial Eco-Restoration in Metropolitan Area, Ministry of Natural Resources, Shanghai 200003, China; 3 School of Finance and Business, Shanghai Normal University, Shanghai 200234, China

**Keywords:** construction land reduction, economic–social–ecological objectives, multivariate probit model, policy acceptance, Shanghai, spatial injustice

## Abstract

Construction land reduction (CLR) is a policy innovation for Shanghai to explore high-quality economic development, but it will also lead to spatial injustice in the implementation process. Although the literature on spatial injustice and CLR is increasing, very little is known about the influence of spatial injustice in CLR on residents’ policy acceptance of the economic–social–ecological objectives of CLR. To fill the knowledge gap, this study uses micro-survey data to identify the factors that influence residents’ policy acceptance of the economic–social–ecological objectives of CLR. Results show that: (1) Spatial injustice in CLR significantly reduces residents’ policy acceptance of the social and ecological objectives of CLR. (2) The locational disadvantage of villages significantly reduces residents’ policy acceptance of the ecological objectives of CLR. (3) The more educated the residents are, the more they recognize the social and ecological objectives of CLR. (4) The higher the percentage of household workers, the more residents endorse the economic and social objectives of CLR. (5) Compared with ordinary residents, cadres are more accepting of the economic objectives of CLR. (6) Robustness tests support the findings of this study. The findings of this study provide insights for sustainable CLR policy reform.

## 1. Introduction

The construction land problems in the process of high-quality development are very comprehensive and far-reaching in Shanghai, China. Land use spatial optimization has drummed up considerable attention [1]. The demand for high-quality development has led to the need to control the expansion of construction land [2]. Construction land reduction (CLR) is a policy innovation, under which high-quality development is explored in Shanghai, China. Shanghai has implemented CLR since 2014. In 2014, JJ Town, W District of Shanghai, took the lead in implementing CLR [3]. CLR is a land restoration tool that reclaims inefficient, dispersed, and heavily polluted construction land outside urban concentrated construction areas into cultivated land or ecological land and generates an equal amount of land quota for construction purposes [2,4,5,6,7,8]. CLR is a development model that helps cope with the constraints of construction land quota [2,3,5]. CLR optimizes the use of construction land and increases the output efficiency of construction land. This is particularly pertinent with the increasing scarcity of construction land quota [4]. Under the premise of the control of the total amount and intensity of construction land, high-quality development in Shanghai should follow the path of CLR [2]. The basic problem with the development of peri-urban regions and remote suburbs is the transfer of land development rights across regions [3]. CLR essentially limits the right to development in a region, which forces the need for high-quality economic development through the optimization of land development rights. Previous studies on CLR have mainly focused on the impact of CLR on towns and villages [4], location choice [2], policy framework or mechanism of life cycle management of industrial land in China [9], and performance evaluation of land consolidation project [10]. It can be found that the studies on CLR mainly focus on the economic and social impact aspects of CLR, and there are fewer studies on the slow development of regions with net reduction of construction land.

CLR may lead to spatial injustice. Soja links justice to other broad concepts referring to the quality of a just society, such as freedom, liberty, equality, etc. [11]. Since Rawl’s concept of distributional justice [12], the distribution of resources has been central also to the geographical aspect of justice [13]. In this study, spatial justice means that according to the principle of equity, the reduction obligation of the subject is equivalent to the obligation of other subjects, or the right to use the land quota obtained from CLR for new construction projects is equivalent [3]. At present, in the process of CLR, it is difficult to give full play to the advantage of backwardness of the remote suburbs and protect their development rights and interests, which lead to spatial injustice. Under the premise of the control of the total amount of construction land, the development potential of the remote suburbs may be weakened; this results in spatial injustice. In terms of the task of CLR, the remote suburbs have more tasks, while in terms of the use of construction land obtained from CLR, the construction land used in the remote suburbs is limited due to low efficiency of construction land. This difference is an important reason for spatial injustice. As far as the relevant literature is concerned, some studies focus on environmental justice [14,15], environmental justice claims [16,17], environmental burden [17], the global environmental justice campaign [18], or hierarchy and inequalities within socially vulnerable groups [19,20], and transition and justice [21]. Other studies focus on spatial inequalities in higher education attainment [22]. Some studies have also analyzed the effect of spatial justice on residents’ policy acceptance of CLR [3]. It can be seen that there are fewer quantitative studies on spatial justice, especially those related to the impact of CLR on the development of disadvantaged regions.

CLR is an important policy with economic–social–ecological objectives. CLR has social and ecological objectives, as well as economic objectives: (1) The economic objectives focus on economic efficiency. Economic objectives are to improve the level of economic development through CLR, not only the growth or speed growth of total gross domestic product (GDP), but also through the optimization of the spatial layout of construction land, improving the mobility of land factor, and increasing the economic output per unit of construction land. As a whole, economic objectives are to improve the total factor productivity. Industrial structure is adjusted to improve economic efficiency and promote high-quality economic development through CLR. This reduces the inefficient and scattered construction land outside the centralized construction area, and allocates the surplus quota to the development of state-owned land in the centralized construction area, so as to create high-quality enterprises. (2) The social objectives focus on social benefits. Social objectives are to promote the development of urbanization. Through urbanization, the structure of residential land can be optimized and the living conditions of residents can be improved. Social objectives include creating more jobs, improving the quality of residents’ employment, and increasing the construction of public facilities and services such as education, medical care and sanitation, etc. Permanently retaining agricultural and forest land in the remote suburbs is a financial challenge due to pressure on metropolitan development [23]. Transferring surplus labor from the agricultural sector to production jobs in the modern urban sector could be considered in the CLR of Shanghai, China [24]. The municipal government of Chengdu, China simultaneously absorbed the surplus labor force of rural areas in the process of economic development, a strategy worthy of consideration by other planners [25]. (3) The ecological objectives focus on ecological benefits. Ecological objectives are to reduce the number of inefficient and seriously polluting enterprises, introduce high-quality enterprises, and centralize the construction land quota to the centralized construction area, so as to reduce the damage to the ecological environment caused by economic and social development. Ecological objectives focus on ecological benefits, which create an agglomeration economy and reduce environmental damage by developing centralized industries [25]. CLR reduces environmental pollution by shutting down highly polluting and economically inefficient businesses. The introduction of high-quality enterprises can also reduce environmental pollution.

Improving residents’ policy acceptance of CLR is of great practical significance for the effective implementation of such a policy. Policy acceptance plays a very important role in policy implementation [26]. Therefore, improving residents’ policy acceptance of the objectives of CLR is of great practical significance when promoting the implementation effect of CLR. However, few previous studies have focused on residents’ policy acceptance of the economic–social–ecological objectives of CLR. Several studies focused on policy acceptance issues, such as the role of landscape identity in policy implementation [27], farmer policy satisfaction levels with the withdrawal of homestead [28], and farmer satisfaction in the policy of increase–decrease linking [29]. Some studies measured the residential satisfaction of residents in six urban renewal projects in Chongqing, China [30]. Some previous studies relate to CLR or the economic objectives of land-use plans [31,32], whilst others focus on the social objectives [33,34]. There are many studies of land-use planning paying attention to the ecological objectives [35]. The city of Wuhan, China has been used by former researchers as the sample to analyze the multi-objective optimization of land use in space and time, and it was found that interest conflicts exist between the differing objectives [36]. However, most existing studies are based on the perspective of land planning technology and do not comprehensively take into account the economic, social, and ecological objectives of CLR. To date, no literature has used a multivariate probit (MVP) model to study CLR and residents’ policy acceptance of the economic–social–ecological objectives of CLR.

Considering the limitations of the above established studies, this study considers how spatial injustice affects residents’ policy acceptance of the economic–social–ecological objectives of CLR. Based on the micro-survey data from a net reduction region of construction land, JJ Town in the W District, Shanghai, China, we empirically studied the impact of spatial injustice on residents’ policy acceptance of the economic–social–ecological objectives of CLR. We also identify the influencing factors that affect residents’ policy acceptance of the economic–social–ecological objectives of CLR. This study serves as a valuable reference for the implementation of CLR in other regions of the rest of China and other developing or developed countries.

The rest of this study is structured as follows. Section 2 analyzes the influence mechanism of spatial injustice on residents’ policy acceptance of the economic–social–ecological objectives of CLR; Section 3 describes the data and methods; Section 4 is the regression results and analysis; Section 5 is the discussion; the last part comprises the conclusions and policy implications.

## 2. Theoretical Framework

### 2.1. Influence of Spatial Injustice on Residents’ Policy Acceptance of the Economic–Social–Ecological Objectives of CLR

The degree of residents’ policy acceptance is an important influencing factor when judging the success of a policy [37]. Public participation is of great significance when formulating and improving government policies. The Central Committee City Work Conference was held in Beijing, China again in December 2015, 37 years later than 1978. It was clearly identified that the relevant provisions on public participation should be conscientiously implemented (See: Opinions on Further Strengthening the Management of Urban Planning and Construction by the Central Committee of the Communist Party of China and the State Council, http://http://www.gov.cn/zhengce/2016-02/21/content_5044367.htm, 2016, accessed on 25 January 2023). The decision-making procedure with democratic participation is both a component and a condition of social justice [38]. Some studies believe it incomplete to define environmental justice as fairness. It should include an equitable distribution of environmental risks, recognition of the diversity of community participants, and the political process involved in formulating and managing environmental policies [18]. The priorities of land planning, competition for land use, environmental degradation, the lack of farmer participation in land development, opaque procedures, and economic barriers, are all manifestations of injustice [39]. Various stakeholders including farmers and other interest objectives have made limited efforts to ensure a long-term and sustainable resolution of land injustice. Additionally, farmers should organize to safeguard their personal various interests and participate in the urban planning and development processes [39].

The key to fairness lies in the equality of social rights and interests. The premise of urban and rural development is that the two should be equal. The inequality of the rights and obligations between the land-reduced regions and the quota-used regions in the process of CLR is the root of spatial injustice in CLR. Agricultural production has ecological value [40]. Suburban agricultural production has the function of ecological environmental production, providing necessary ecological products and services for human survival. Such services and the natural capital stock that generate such services are essential to the functioning of Earth’s life support systems [41]. China’s “Central Committee No.1 Document” (2018) clearly specified the need for an “increase in the supply of agricultural ecological products and services”. In 2015, the construction of JJ Country Park in JJ Town, W District, Shanghai, has the nature of public welfare. Whilst the Country Park will drive some tourism for the economy, the ecological benefits obviously outweigh the economic benefits. JJ Country Park helps to deal with the shortage of green space in built-up areas, and to meet the increasing demands by residents for outdoor amenities due to rapid urbanization [42]. Previous research underlines that land consolidation is an important way to improve ecological and environmental quality [43,44,45].

JJ Town is located in the suburbs of Shanghai, and is a net reduction region of construction land according to the city plan and CLR. It is a less-developed region, which is facing a series of land problems [46], and where economic development lags far behind the peri-urban areas. JJ Town is a region of net outflow of population to cities and towns, where there is a concentration of living space and ecology. As agriculture has ecological value, Shanghai gives much ecological compensation for agricultural land. For example, according to the Notice on the Basic Farmland Ecological Compensation Funds (No. 112, 2018) by Shanghai Minhang District Agriculture Commission and Shanghai Minhang District Finance Bureau, the 2018 ecological compensation standard for basic farmland was CNY 1500+/mu (1 mu ≈ 0.067 hectares), half of which is used for village public service expenditure and the other half is directly given to farmers. Agriculture plays a role in public welfare and produces agricultural products that have ecological function. The government affirms the ecological function of agriculture and provides ecological compensation for agricultural land. Therefore, they have a higher degree of policy acceptance of the ecological objectives of CLR. In general, the economic output of agricultural land is relatively low, meaning that resident’s policy acceptance of the economic objectives of CLR is relatively weak. Some residents in suburban areas have less contracted land, but as the government’s ecological compensation is based on the contracted land, the income from the contracted land accounts for a small proportion of resident total income. Moreover, residents in Shanghai, China are generally disengaged in agricultural production, and have already decoupled from the land. The existing centralized residential policy is based on the homestead, which means residents with homesteads will have the opportunity to concentrate their residence and use city resources to achieve a better quality of life than in the rural areas.

To this end, Hypothesis 1 is proposed:

 **Hypothesis 1 (H1).** *Spatial injustice leads to the unobvious decrease in residents’ policy acceptance of the economic objectives of CLR, leading to an obvious decrease in residents’ policy acceptance of social and ecological objectives of CLR. In other words, the higher the realization of spatial justice in CLR, the more attention residents attach to the social and ecological objectives of CLR rather than the economic objectives of CLR*.

### 2.2. Influence of Spatial Injustice on Cadres’ Policy Acceptance of the Economic–Social–Ecological Objectives of CLR

Spatial injustice is rooted in the unequal spatial allocation of land development rights in the CLR process. The behavior of cadres can be directly influenced by the performance assessment of CLR by the higher levels of government. The low elasticity of non-agricultural construction land supply and the pressure of performance assessment make the economic growth model based on traditional land finance unsustainable. In economically developed regions of China, CLR has become an innovative path for high-quality economic development. Under the assessment and accountability mechanism, cadres’ performance will be included in their annual assessment. Those cadres who do not pay enough attention to CLR and/or implement inadequate measures, and are slow to rectify the situation will be interviewed [47,48]. The higher-level government wants to expand the radius of CLR to optimize the allocation of construction land quotas so as to achieve sustainable development [5]. As a result, the development space for regions of less efficient construction land will continue to shrink and, therefore, their development will progressively slow down. Subsequently, the performance level of cadres will continue to decrease. Cadres in different regions have different views on performance assessment. The performance assessment system has created constraints on cadres. Pudong New Area of Shanghai, China has implemented a cadre assessment system that combines daily and annual assessments in order to improve the accuracy and credibility of cadre assessments. At the same time, the performance assessment basis of positive incentive and negative accountability provides a basis for the “combination of assessment and appointment” [49]. The cadres in different townships (villages), especially those in the same district, will frequently communicate with each other. In the CLR process, cadres will compare with each other, including the scale, distribution, and progress of CLR, etc. Stakeholders in the CLR process include municipal government, district governments, township governments, land enterprises, residents, and village collectives [5]. The main body for implementing CLR under spatial planning is the township government. The government obtains revenue from the incremental construction land and extracts a portion of it for compensation to the relevant interest subjects. Thus, the collective interests of the village have suffered a significant loss. For instance, the reduction of construction land leads to a reduction of development space and long-term rental income [4].

Rental income may face a sharp decline or even disappear after CLR. Unlike the traditional policy of increase–decrease linking, CLR in Shanghai does not require closure in the operation of the project. The rural collective economic organizations do not directly participate in the distribution of the land appreciation proceeds corresponding to CLR, but are mainly compensated for losses indirectly by the higher-level government. The reclamation of agricultural land generated by CLR is an important part of Shanghai’s implementation of the spirit of the 19th National Congress of the Communist Party of China and the green development concept of “clear waters and green mountains are as good as mountains of gold and silver”. For CLR, Shanghai has established a whole life cycle management model for land consolidation and reclamation projects. Therefore, CLR in Shanghai, China, has obvious government-led characteristics. The Shanghai municipal government has constrained local governments through administrative pressure to motivate district and township governments to carry out CLR tasks in accordance with the necessary requirements [5].

In practice, township governments lack the incentive to implement CLR. Governments in remote suburbs are still using low-priced industrial land to attract investments to increase tax revenues. Their urbanization and industrial development still depend on land financing, and thus CLR weakens their revenue capacity, especially if the remaining quota of CLR is not used for their own new construction projects. The towns (villages) rely on subsidies and quota acquisition fee from higher levels of government to balance the cost of CLR, so they are not very motivated to implement CLR. Thus, spatial injustice can have an impact on the assessment of cadres, acting as both a positive incentive and a negative accountability. The performance assessment by the higher-level government of the lower-level government includes both the GDP assessment of the year and the assessment of the CLR’s task completion [5]. The assessment of GDP is relatively simple and straightforward [5]. In the CLR region, there is the assessment of GDP and the task of CLR. In the non-CLR regions, there is no assessment of the task of CLR. Thus, for CLR regions, cadres have the motivation to develop the economy under the pressure of assessment and the need to complete the task of CLR. However, this may be related to the type of CLR. Regions with net increase in construction land have fewer CLR tasks and may focus more on GDP growth. In contrast, regions with net reduction in construction land, with poor construction land efficiency and more CLR tasks, may focus more on the completion of CLR tasks. In addition, the social and ecological objectives of CLR are difficult to assess and lack a systematic assessment mechanism. As a result, the acceptance of the social and ecological objectives of CLR by cadres may be similar to that of ordinary residents.

In view of this, Hypothesis 2 is proposed:

 **Hypothesis 2 (H2).** *Under the pressure of performance assessment, cadres are more receptive to the economic objectives of CLR than ordinary residents, while their receptiveness to the ecological and social objectives of CLR is similar to that of ordinary residents*.

## 3. Data and Methods

### 3.1. Research Area

A villager group (production team) collective in rural China is a fully autonomous social organization. CLR in Shanghai, China is implemented with the villager group as the basic unit [3]. Therefore, this study takes the villager group as the basic research unit. In 2014, JJ Town, W District of Shanghai, took the lead in implementing the policy of CLR to pursue the high-quality use of land [3]. JJ Town represents a category of net reduction region for construction land and, at the village level, there are both regions of net incremental and net reduction of construction land. This is the geospatial unit and basis for the spatial injustice analysis. At the village level, JJ Town can represent the actual policy practice of CLR in Shanghai, China to a certain extent. As a result, the research group selected JJ Town, W District, Shanghai, as the research site. JJ Town is located in the middle-southwest of W District, Shanghai, China. The development planning of JJ Town concerns a rapid urbanization where the population should be transferred to urban areas. There is a modern agricultural park at the municipal level in JJ Town, W District, Shanghai, China. It is a comprehensive agricultural park with economic, ecological, and social benefits. This also enhances resident’s policy acceptance of the ecological objectives of CLR.

### 3.2. Survey Implementation

Data were obtained through interviews and questionnaires with residents of 11 administrative villages and two village committees from September to October 2020. Four hundred questionnaires were distributed, and 344 valid questionnaires were collected, including 306 local resident questionnaires, with an effective recovery rate of 86%. Remote sensing image data was processed by ArcGIS to extract construction land data. The gender ratio of respondents is nearly 1:1. In terms of age distribution, the number of 31–45 years is the largest, accounting for 33.99%, followed by 45–60 years, accounting for 31.37%. From the perspective of education distribution, most are of lower secondary school, accounting for 35.29%, followed by college and above, which account for 31.70%. In terms of resident status, ordinary residents (non-cadre) account for the largest proportion, at 80.07%. In terms of household size, those with five members account for 43.14%, followed by four member families, accounting for 17.97%. As for contracting land scale, 0–2 mu has the least respondents, accounting for 30.07%. With regards to household income, 100,000 CNY–200,000 CNY account for 40.52%, followed by 50,000 CNY–100,000 CNY, which accounts for 25.16%.

**Figure 1:**
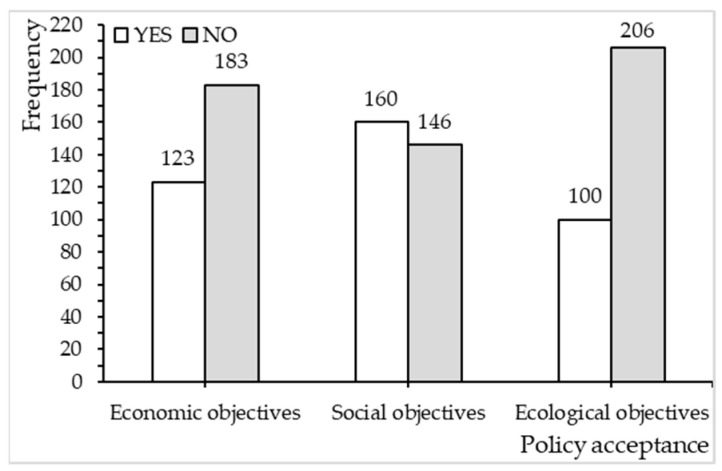
Residents’ policy acceptance of the economic–social–ecological objectives of CLR. Source: authors’ own work.

Figure 1 shows residents’ policy acceptance of the economic–social–ecological objectives of CLR. It can be seen that respondents are in greater agreement with the social and economic objectives of CLR than the ecological objectives of CLR.

### 3.3. Model Selection

CLR is a model innovation of sustainable development [2,5], which guides high-quality economic and social development. It has three main policy objectives: economic objectives; social objectives, and ecological objectives. Residents’ policy acceptance of the objectives of CLR is also multidimensional and has certain correlations between their choice of these policy objectives. The choice between different policy information sources of CLR is not mutually exclusive, making the random error terms likely to be interrelated [50]. Neither the regression model using simple binomial Logit, nor the model using polynomial Logit, can explain the problem of multiple dependent variables. Simple binomial Logit and the model using polynomial Logit also not conducive to cross-sectional comparison of multiple decisions. As there is an intercorrelation amongst residents’ choice of the economic–social–ecological objectives of CLR, joint estimates of the above three policy objectives are needed. Since the MVP model allows for a correlation amongst the error terms of different equations [50,51], we improved the accuracy of our estimated results by using previous research on MVP models to explore the choice behavior of residents on the different policy objectives of CLR.

The MVP model can be used in a general form to simultaneously handle multiple binary choices [52], as shown below:

y_im_* = *β_m_′X_im_* + *ε_im_*, *m* = 1, …, M (1)

The dependent variables satisfy below:

*y_im_* = 1 if y_im_* > 0; *y_im_* = 0 otherwise (2)

whereby *i* refers to the *i* resident. *ε_im_* (*m* = 1, …, M) is the random error item that obeys multivariate normal distribution. *y_im_* = 1 and *y_im_* = 0 refers to the number of *i* residents that do or do not choose the *m* policy objective, respectively. M refers to the number of M policy objectives. *X_im_* refers to the following factors affecting residents’ choice of the policy objectives: spatial injustice; compensation standard; village location disadvantage; gender; age; level of education; household income; contracting land scale; household population structure. Estimates can be obtained by conducting maximum likelihood fitting estimates to Equation (1).

**Figure 2:**
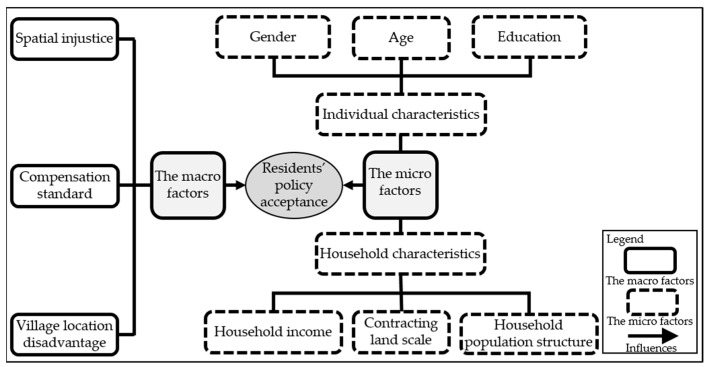
The empirical analysis framework of this study. Source: authors’ own work.

The empirical analysis framework of this study is shown in Figure 2.

### 3.4. Variable Selection and Index Measurement

#### 3.4.1. Dependent Variables

The dependent variable is residents’ policy acceptance of the economic–social–ecological objectives of CLR. These include residents’ policy acceptance of the economic objectives (*Y*_1_), the social objectives (*Y*_2_), and the ecological objectives (*Y*_3_) of CLR. In particular, based on the characteristics of CLR, this study explains the three objectives of CLR from three types of land: industrial land, residential land, and ecological land. The idea is: the focus of CLR is industrial land; residential land is also the content of CLR, but it is difficult to promote. Although commercial land will also affect the realization of economic objectives, it is not the key reduction object of CLR. Through CLR, inefficient construction land outside the centralized construction area is reclaimed into cultivated land or ecological land. Therefore, economic objectives can be measured from the perspective of industrial upgrading of industrial land, social objectives can be measured from the perspective of structural optimization of residential land, and ecological objectives can be measured from the perspective of increasing ecological land use and improving ecological land use structure.

#### 3.4.2. Core Explanatory Variable

The core explanatory variable of this study is spatial injustice. Due to the difference in population and construction land bases in different villages, the net increase of construction land cannot reflect the degree of realizing the construction land rights and interests of each village. Therefore, this study measures the spatial injustice from the dual perspective of per capita spatial injustice and per land spatial injustice. The increase or decrease in construction land is the geospatial unit and basis of spatial injustice analysis in this study. According to the principle of equity, the reduction obligation of the subject is equivalent to the obligation of other subjects. However, the reality of CLR is that there is a net decrease in construction land quota in net reduction regions and a net increase in construction land in net increase regions. This difference, i.e., the amount of construction land allocation, reflects to some extent the difference in the development space of construction land. In this study, we measure the spatial injustice from the perspective of the allocation of construction land quotas, i.e., the realization of such spatial rights and interests as the development space of construction land.

(a) Per Capita Spatial Injustice

Spatial injustice can be measured based on the perspective of the realization of per capita rights and interests on the construction land. On the one hand, population size can reflect the level of demand for the number and type of life services in an area [53,54,55]. On the other hand, per capita land size can indicate the quantitative relationship between the population of a given area and the life service opportunities provided [56], and this quota is also used by urban planners to identify the scarcity of the land available for each type of service, based on minimum service standards [55]. Therefore, as an important carrier of public services, the construction land partially reflects the supply of public services in a region, and the demand of public services reflects the population size. The spatial justice of CLR should ensure the fair allocation of the net increment of per capita construction land in all regions, so as to ensure the total welfare of the CLR regions without reducing the per capita welfare.

Therefore, this study carries out the measurement based on the realization of per capita construction land rights and interests, with reference to the assignment method of income quantile [57]. CLR in Shanghai has been fully implemented since 2014, and it has also been included in a new round of CLR (2017 to 2035). In relation to the data collection, the distribution of the net increment of per capita construction land for the registered population from 2013 to 2018 is used as a proxy variable:

*CLPNI_i,_*_2013_*_–_*_2018_ = (*CL_i,_*_2018_ − *CL_i,_*_2013_)/*HJP_i_*_,2013_ (3)

In Equation (3), *CLPNI_i_*_,2013–2018_ refers to the net increment in per capita construction land corresponding to the registered population from 2013 to 2018 of the *i* village. *CL_i_*_,2018_ and *CL_i_*_,2013_ refer to the construction land size of the *i* village in 2018 and 2013, respectively. *HJP_i_*_,2013_ refers to the registered population scale of the *i* village in 2013. According to the sample size of this study, the net increment of the scale of per capita construction land is ranked from lowest to highest and grouped by quartile, in order to measure the per capita spatial injustice (*SIRJ*). The higher the net increment of the per capita construction land scale, the higher the realization of the construction land rights in the area, and the weaker the per capita spatial injustice, meaning greater spatial justice. According to the Master Plan and General Land Use Plan of W District, Shanghai (2017–2035), from the planning base year (the end of 2016) to 2035, the construction land area of JJ Town will decrease by 15 percentage points. The space for construction land is extremely limited under the control of total amount and intensity of construction land. Therefore, in areas with small use of construction land, development space is compressed, which belongs to the category of spatial injustice [3].

(b) Per Land Spatial Injustice

Since the construction land area of each village is different, we also re-measure spatial injustice from the perspective of per land spatial injustice. Spatial injustice has a regional theoretical connotation, which essentially reflects the spatial allocation of the total social welfare. Construction land provides public service functions, such as employment and residence, and is the material carrier of social welfare. The lower the net increment of construction land in a region, the less conducive it is to improving the total social benefits in the region. Spatial justice of CLR ensures the balanced distribution of total social benefits in all regions. Therefore, this study carries out the measurement of spatial injustice based on the realization of per land construction land rights with reference to the assignment method of income quantile [57]. With regards to the data collection, this study uses the distribution of the net increment of the construction land that corresponds to the unit construction land from 2013 to 2018 as a proxy variable:

*CLLNI_i_*_,2013–2018_ = (*CL_i_*_,2018_ − *CL_i_*_,2013_)/*CL_i_*_,2013_ (4)

In Equation (4), *CLLNI_i_*_,2013–2018_ refers to the net increment in per land construction land corresponding to the unit construction land from 2013 to 2018 of *i* village. *CL_i_*_,2018_ and *CL_i_*_,2013_ are consistent with Equation (3). With regards to the sample size of this study, the net increment of the scale of per land construction land is ranked from lowest to highest and grouped by quartile, in order to re-measure the per land spatial injustice *(SIDJ)*. The higher the net increment of the per land construction land scale, the higher the realization of the construction land rights and interests in the area, and the weaker the per-land spatial injustice, thus reflecting a strong spatial justice of CLR.

#### 3.4.3. Other Explanatory Variables

In reference to [29,44], we select the following explanatory variables, which we then combine with research reality: compensation standard (*CS*); village location disadvantage (*VLD*); gender (*GEN*); age (*AGE*); level of education (*EDU*); household income (*HI*); contracting land scale (*CLS*); household population structure (*HPS*). During the implementation of CLR, the higher-level government will provide certain subsidies to lower-level governments in order to mobilize enthusiasm amongst residents and cadres within the towns. These subsidies mainly comprise economic compensation, with insufficient attention paid to ecological compensation. The landowner’s cognitive behavior helps explain their motivation towards land consolidation and other related projects [45]. Therefore, the degree of the compensation standard for CLR partially reflects the extent to which the government protects the rights of reduction regions. The more reasonable the compensation standard, the stronger the resident’s policy acceptance is. *VLD* is measured by the distance from the village to the government station of JJ town. The greater the distance value, the weaker the location advantage. Villages with a lower distance value possess better location conditions, have higher demand for the optimization of the construction land structure, and more strongly support the CLR. The personal characteristics of residents include *GEN*, *AGE*, and *EDU*. Household characteristics include *HI*, *CLS*, and *HPS* of 2019. The resident status variable (*GB*) is added for heterogeneity analysis of resident status. A specific variable explanation and index measurement of the model are shown in Table 1. Descriptive statistics of each index are shown in Table 2.

## 4. Results

### 4.1. Baseline Regression Results

In the baseline regression, residents’ policy acceptance of the economic–social–ecological objectives of CLR are used as the dependent variables, and *SIRJ* is the core independent variable. *CS*, *VLD*, *GEN*, *AGE*, *EDU*, *HI*, *CLS*, and *HPS* as other considered variables. The number of draws is set to 18 with the reference of related literature [58]. The principle is obtaining the robust regression results by setting the number of random draws slightly greater than the arithmetic square root of the sample size. The results of the MVP model regression are shown in Column (1)–(3) of Table 3. In model (1)–(3) of Table 3, the regression likelihood ratio test for the dependent variable of the MVP model showed a chi-square value of rho21 = rho31 = rho32 is 36.3245, with a significance probability *p*-value of 0.0000. Thus, the original hypothesis that the correlation coefficient of the dependent variable is 0 is rejected, indicating the validity of using the MVP model.

Columns (1)–(3) of Table 3 detail the regression results for all residents. It can be seen that resident opinions of the three core objectives of CLR are interrelated. It is also evident that per capita spatial injustice significantly reduces the degree of residents’ recognition of the social and ecological objectives of CLR.

Spatial injustice significantly reduces residents’ policy acceptance of the social and ecological objectives of CLR. Social objectives aim to improve the improvement in people’s livelihood by means of social development, such as urbanization and concentrated housing. JJ Town is a net reduction region of construction land in the W District, and the outflow of construction land quota is not conducive to the urbanization of the town, nor does it provide sufficient quota for centralized living. Therefore, spatial injustice significantly weakens residents’ policy acceptance of the social objectives of CLR. In other words, improving the spatial justice of CLR helps strengthen residents’ policy acceptance of the social objectives of CLR. Therefore, Hypothesis H1 has been verified.

Why does spatial injustice significantly reduce residents’ policy acceptance of the ecological objectives of CLR? There exist several possible explanations: (1) The metropolitan city has been transformed from a production city to a consumer city, with relatively high requirements for urbanization, concentrated living, and ecological environmental improvement, and the residents favor ecological development. (2) Ecological benefits come from environmental management which provides ecological benefits, such as rivers, greening, and country parks. As a core city of the Yangtze River Delta urban agglomeration, Shanghai already has outstanding social development advantages in terms of human resources, and the pursuit of high-level talents has enhanced the need for improvement in the ecological environment. (3) Shanghai has explored reducing the development of construction land, focusing rather on improving the social and ecological, rather than economic, benefits. This has been one of Shanghai’s main achievements with regards to promoting ecological civilization (Ecological civilization is a social form with the basic purpose of harmonious coexistence, virtuous cycle, all-round development and sustained prosperity between man and nature, man and man, and man and society. Ecological civilization pays more attention to the protection of the ecological environment and reduces the negative impact of human production and life on the ecological environment.) since the 18th Communist Party of China National Congress in 2012. JJ Town also urgently needs to improve the ecological environment and reduce environmental pollution. According to the 2019 Statistical Yearbook of W District, the energy consumption of industrial land in 2018 was 16,203.78 (tons of standard coal), an increase of 5.88% when compared with 2017. Therefore, under the mode of reduction development, spatial justice of CLR strengthens residents’ policy acceptance of the social and ecological objectives of CLR, whilst their acceptance of economic objectives of CLR is not obvious.

With regards to compensation standard, the rationale of compensation standard significantly enhances residents’ policy acceptance of economic objectives of CLR. This is because the compensation object is mainly economic loss and the compensation standard is low, with insufficient attention paid to ecological compensation. So, the insufficient attention to ecological compensation, and the compensation object being economic loss, significantly reduce residents’ policy acceptance of the ecological objectives of CLR. The locational disadvantage of villages significantly reduces residents’ policy acceptance of the ecological objectives of CLR. The older the residents are, the higher their acceptance of the social objectives of CLR is. The more educated the residents are, the more they recognize the social and ecological objectives of CLR. The residents with higher household income have higher acceptance of the economic objectives of CLR. In Shanghai, China, the government affirms the ecological function of agriculture and provides ecological compensation for agricultural land. The government ecological compensation is based on the contracted land scale. Therefore, residents with more contracting land are more receptive to the ecological objectives of CLR.

Regarding household population structure, the higher the proportion of family working population, the stronger the acceptance of the economic and social objectives of CLR. According to the 2019 Statistical Yearbook of W District, residents working at the primary agricultural industry in JJ Town accounted for only 7.69% in 2018, whilst the vast majority were employed in the non-agricultural sector. In 2019, the total agricultural output value of W District accounted for only 2.47% of its total output value. Subsequently, with an increased proportion of the working family population, greater family social mobility, a larger demand for economic development and living condition improvement, the family members become more competitive in the job market. This makes it easier for them to live in the city during the economic structure optimization process through CLR. Therefore, one can conclude that the greater the proportion of working population in a household, the greater the acceptance of the economic and social objectives of CLR.

### 4.2. Heterogeneity Analysis

Given the heterogeneity of resident status, this study further introduced the resident status variable. Residents’ policy acceptance of the economic–social–ecological objectives of CLR are used as the dependent variables; *SIRJ* and *GB* were the core independent variables; *CS*, *VLD*, *GEN*, *AGE*, *EDU*, *HI*, *CLS*, and *HPS* were used as other variables. In reference to previous literature [58], the number of draws is set to 18. The results of the MVP model regression are shown in Columns (4)–(6) of Table 3. In models (4)–(6) of Table 3, the regression likelihood ratio test for the dependent variable of the MVP model showed a chi-square value of rho21 = rho31 = rho32 is 37.0184, with a significance probability p-value of 0.0000. Thus, this indicates the validity of using the MVP model.

Columns (4)–(6) of Table 3 show the regression results after adding the resident status variable. In comparison to ordinary residents, cadres have a higher acceptance of economic objectives of CLR. This indicates that the existing CLR performance assessment makes cadres focus more on economic development. The economic level of JJ Town is lower than that of other towns in W District, making the demand for economic development more urgent. On the premise of completing the task of reduction, the government also hopes to develop the local economy through industrial transformation and upgrading, as well as land use structure optimization. Since JJ Town belongs to the net reduction region of construction land in the remote suburbs, the net outflow of construction land quota has a negative impact on local economic development. According to the *2019 Statistical Yearbook of W District*, the output value of industrial land in 2018 was about CNY 2.32 billion, a slight increase compare to 2017. However, when compared with other towns in W District, both the base number and the relative amount of construction land were smaller, which limits the economic development of JJ Town. Due to CLR, the original inefficient construction land was reduced. The new construction land quota outflow also restrains the economic development of JJ Town. Therefore, the local cadres recognize economic objectives more than ecological and ecological objectives of CLR. Research Hypothesis H2 is verified.

### 4.3. Robustness Test

Using the baseline regression results, we re-measured spatial injustice from the perspective of the net increment of per land construction land scale, and we conducted a robustness test to the regression results. Residents’ policy acceptance of the economic–social–ecological objectives of CLR are used as the dependent variables; *SIDJ* is used as the core independent variable; *CS*, *VLD*, *GEN*, *AGE*, *EDU*, *HI*, *CLS*, and *HPS* are used sas other considered variables. The number of draws is set to 18 with the reference of related literature [58]. The MVP model regression results are shown in Columns (1)–(3) of Table 4. It can be seen that spatial injustice significantly reduces residents’ policy acceptance of the social and ecological objectives of CLR. The greater the spatial justice, the greater the residents’ policy acceptance of the social and ecological objectives of CLR. In model (1)–(3) of Table 4, the regression likelihood ratio test for the dependent variable of the MVP model showed a chi-square value of rho21 = rho31 = rho32 is 37.2970 with a significance probability p-value of 0.0000. Thus, indicating the validity of using the MVP model.

Considering the heterogeneity of resident status, residents’ policy acceptance of the economic-social-ecological objectives of CLR are used as the dependent variables; *SIRJ* and *GB* were the core independent variables; *CS*, *VLD*, *GEN*, *AGE*, *EDU*, *HI*, *CLS*, and *HPS* were used as other variables. In models (4)–(6) of Table 4, the regression likelihood ratio test for the dependent variable of the MVP model showed a chi-square value of rho21 = rho31 = rho32 is 38.0422, with a significance probability p-value of 0.0000. This indicates the validity of using the MVP model. The MVP model regression results are shown in Columns (4)–(6) of Table 4. Compared to ordinary residents, cadres have a higher acceptance of economic objectives of CLR.

Therefore, the findings of this study are robust.

## 5. Discussion

This study theoretically analyzes how spatial injustice in CLR affects residents’ policy acceptance of the economic–social–ecological objectives of CLR, and conducts an empirical study using micro-survey data. The marginal contributions of this study include: (1) This study explains the theoretical connotation of spatial injustice in CLR, and analyzes how spatial injustice affects residents’ policy acceptance of the economic–social–ecological objectives of CLR. (2) Other factors influencing residents’ policy acceptance of the economic–social–ecological objectives of CLR are identified. (3) This study investigates the heterogeneous impact of resident status on residents’ policy acceptance of the economic–social–ecological objectives of CLR. (4) The policy implications for correcting spatial injustice in CLR and improving residents’ policy acceptance of the economic–social–ecological objectives of CLR are proposed.

CLR involves the reallocation of the construction land surplus quota [2]; in essence, it is the reallocation of the right to change land use to achieve the development objectives. Due to the fixation of land location, this reallocation of land quota is essentially the reallocation of land development rights [59]. Such reallocation of land quota is not unique to Shanghai, China, as it has been used in other economically developed countries. For example, the USA’s development right transfer plan was a multi-objective policy tool implemented by planners to achieve a wide range of planning objectives [59,60]. By 2016, the USA’s local governments had implemented 254 development right transfer projects in 36 states [59]. Scarcity of construction land quota has been an important constraint for economic and social development [4,61,62]. CLR has become a way to solve the contradiction between supply and demand of construction land. Although other cities are exploring CLR policies one after another, Shanghai is one of the most mature provincial cities in terms of CLR policies. This study uses micro-survey data from JJ Town in W District, Shanghai to investigate how spatial injustice in CLR affects residents’ policy acceptance of the economic–social–ecological objectives of CLR. It is an extension of the existing research on CLR. Consistent with the established literature [3,5], this study emphasizes the importance of securing development rights in regions with net reduction of construction land. As the established literature points out, location is an important factor influencing CLR [2,5]. This study finds that location disadvantage significantly reduces residents’ policy acceptance of the ecological objectives of CLR. Protecting the development rights and interests of net reduction regions requires increasing the compensation rates and expanding the scope of compensation for net reduction regions [5], and the reasonableness of compensation standard significantly enhances residents’ policy acceptance of the economic objectives of CLR. Related studies found that the reasonableness of compensation standard strengthens residents’ preference for the direct economic compensation type scheme [5], which is consistent with the conclusion in this study.

This study found that cadres attached more importance to the economic objectives of CLR, while the acceptance of social and ecological objectives was not significantly different from that of the general residents. In the process of CLR, cadres are the propagators and implementers of the CLR policy; therefore, the cognition of cadres and their acceptance of the economic–social–ecological objectives of CLR are very critical. This study has important implications for the optimization of cadres’ assessment mechanism in the process of CLR.

It is worth noting that spatial justice is a complex concept. Its quantitative measurement is a complex issue, and this study is only an exploration based on the difference in the amount of spatial allocation of construction land from the perspective of the realization of development rights of construction land. If the destination of construction land quotas after CLR in each village can be obtained, it will be important to optimize the measurement of spatial injustice of CLR. Our micro-survey data of JJ town covered every village, which increased the difficulty of expanding the sample to some extent. A subsequent expansion of the study area would help to obtain richer research findings. Nevertheless, this study reflects the residents’ awareness of the CLR policy in the region of net reduction of construction land, which has certain policy implications for the economically developed regions to improve the construction land policy and guarantee the spatial justice of construction land allocation.

## 6. Conclusions and Implications

### 6.1. Conclusions

On the basis of theoretical analysis, the MVP model is established with the micro-survey data of a net reduction region of construction land in the remote suburbs of Shanghai, China. It empirically tests how spatial injustice in CLR affects residents’ policy acceptance of the economic–social–ecological objectives of CLR. Results are as follows:

- Spatial injustice in CLR will significantly decrease residents’ policy acceptance of the social and ecological objectives of CLR, but will not weaken their acceptance of the economic objectives of CLR.
- From the macro perspective, the more reasonable the compensation standard is, the higher the residents’ policy acceptance of the economic objectives of CLR is, and the lower the acceptance of the ecological objectives of CLR is; the location disadvantage of the village has significantly reduced the residents’ policy acceptance of the ecological objectives of CLR.
- From the micro level, residents with higher household income and higher proportion of family labor population have higher acceptance of the economic objectives of CLR; the older the residents are, the higher their education level is, and the higher the proportion of family labor population is, the higher their acceptance of the social objectives of CLR is; the more educated residents are, the higher their acceptance of the ecological objectives of CLR is.
- The heterogeneity analysis shows that cadres are more receptive to the economic objectives of CLR than ordinary residents, while their receptiveness to the ecological and social objectives of CLR is similar to that of ordinary residents.
- The research conclusion of this study is still robust after changing the measurement method of spatial injustice.

### 6.2. Implications

Based on the research conclusions of this study, seven policy implications are obtained:

- CLR is currently an effective way to achieve high-quality sustainable development when the total amount and intensity of construction land is strictly constrained. Therefore, it is necessary to optimize the structure of construction land and improve the efficiency of land use to meet the needs of new construction land for sustainable development.
- It is important to pay attention to the impact of the realization of spatial justice in order to improve residents’ policy acceptance of the economic–social–ecological objectives of CLR. Firstly, increasing the retention proportion of construction land quota in the net reduction regions, and improving land use efficiency through regional industrial structure optimization, are both required to enhance regional economic competitiveness. Secondly, if the construction land quota in the remote suburbs is to be used in peri-urban regions, the peri-urban regions should encourage residents from remote suburbs to work and live in their region, so as to reduce the per capita negative impact of CLR, and protect the interests of the net reduction regions. Thirdly, in the process of transferring construction land quota, it is necessary to increase the compensation for the net reduction regions. This can improve the support of local residents for the transfer out of construction land quota, and enhance their acceptance and satisfaction with the economic–social–ecological objectives of CLR.
- CLR has significant ecological and social benefits, and is conducive to optimizing the regional industrial structure and improving overall economic efficiency for the whole society. Therefore, in the process of CLR, the following are necessary: full attention should be given to the contribution of the net reduction regions; economic compensation standards for such regions should be increased; the scope of economic and cross-regional economic compensation should be expanded.
- In addition to compensating for economic losses in regions with net reduction of construction land, the following are required: improvements in living conditions in densely populated regions, greater employment opportunities, and diversified compensation methods.
- Great importance must be attached to the ecological benefits brought by CLR through improving the compensation standard and expanding the scope of compensation for ecological benefits.
- Greater publicity and the provision of information to residents are necessary to enhance both their policy acceptance of the economic–social–ecological objectives of CLR and their overall satisfaction with government planning policies.
- It is necessary to optimize the cadre assessment mechanism. Compared with the social and ecological objectives of CLR, the cadres of local government departments pay more attention to the economic objectives of CLR. The economic objective is an important one of CLR’s multiple objectives, but not the most important. Under the background of ecological civilization and high-quality development, residents’ employment and ecological environmental protection are more important. Therefore, it is necessary to optimize local government performance assessment, weaken the assessment of GDP of CLR, and strengthen the assessment of the completion of social and ecological objectives of CLR.

## Figures and Tables

**Table 1:** Model variable explanation and index measurement.

Variable Type	Variable Name	Variable Code	Index Measurement
Dependent variables	Residents’ policy acceptance of the economic objectives of CLR	*Y*_1_	Whether it is an effective means to realize the upgrading of domain industries (industrial land). Yes = 1; No = 0.
	Resident’s policy acceptance of the social objectives of CLR	*Y*_2_	Whether is an important means to optimize the structure of residential land (residential land). Yes = 1; No = 0.
	Resident’s policy acceptance of the ecological objectives of CLR	*Y*_3_	Whether is an important means to achieve environmental improvement within the domain (increasing ecological land, improving ecological land structure). Yes = 1; No = 0.
Core explanatory variable	Per capita spatial injustice	*SIRJ*	Quartile value of *CLPNI_i,_*_2013–2018_: The first quartile = 4; the second quartile = 3; the third quartile = 2; the fourth quartile = 1.
	Per land spatial injustice	*SIDJ*	Quartile value of *CLLNI_i_*_,2013–2018_: The first quartile = 4; the second quartile = 3; the third quartile = 2; the fourth quartile = 1.
Other explanatory variables	Compensation standard	*CS*	Residents’ comment on the rationality of the compensation standard for CLR in this town: very reasonable = 5; relatively reasonable = 4; generally reasonable = 3; relatively unreasonable = 2; very unreasonable = 1.
	Village location disadvantage	*VLD*	The distance from the village to the government station of JJ town (km).
Personal characteristic	Gender	*GEN*	Dummy variable. Male = 1; female = 0.
	Age	*AGE*	30 and below = 1; 31–45 = 2; 45–60 = 3; 60 and above = 4
	Level of education	*EDU*	Primary school and below = 1; Lower secondary school = 2; Upper secondary school = 3; college and above = 4
Household characteristic	Household income	*HI*	50,000 CNY and below = 1; 50,000 CNY–100,000 CNY = 2; 100,000 CNY–200,000 CNY = 3; 200,000 CNY and above = 4.
	Contracting land scale	*CLS*	0 mu = 0; 0–0.5 mu = 1; 0.5–1 mu = 2; 1–1.5 mu = 3; 1.5–2 mu = 4; 2 mu and above = 5.
	Household population structure	*HPS*	The proportion of the population aged 18 to 60 years in the total household size.
Heterogeneous variable	Resident status	*GB*	Yes = 1; No = 0

Source: compiled by the author.

**Table 2:** Descriptive statistics of the variables.

Variable	Obs	Mean	Std. Dev	Min	Max
*Y*_1_	306	0.4020	0.4911	0.0000	1.0000
*Y*_2_	306	0.5229	0.5003	0.0000	1.0000
*Y*_3_	306	0.3268	0.4698	0.0000	1.0000
*SIRJ*	306	2.4118	1.1708	1.0000	4.0000
*SIDJ*	306	2.4967	1.0992	1.0000	4.0000
*GB*	306	0.1993	0.4002	0.0000	1.0000
*CS*	306	3.5261	1.0625	1.0000	5.0000
*VLD*	306	3.2669	1.3916	0.0000	5.9553
*GEN*	306	0.5392	0.4993	0.0000	1.0000
*AGE*	306	2.5065	0.9726	1.0000	4.0000
*EDU*	306	2.6895	1.0613	1.0000	4.0000
*HI*	306	2.6307	0.9603	1.0000	4.0000
*CLS*	306	2.5556	2.1876	0.0000	5.0000
*HPS*	306	0.6380	0.2407	0.0000	1.0000

Source: compiled by the author.

**Table 3:** MVP model standard regression results.

Variable	(1)	(2)	(3)	(4)	(5)	(6)
	*Y*_1_	*Y*_2_	*Y*_3_	*Y*_1_	*Y*_2_	*Y*_3_
*SIRJ*	−0.0245	−0.1761 ***	−0.1613 **	−0.0287	−0.1782 ***	−0.1639 **
	(0.0688)	(0.0668)	(0.0728)	(0.0692)	(0.0666)	(0.0729)
*GB*				0.6622 ***	0.0397	0.3340
				(0.2171)	(0.2163)	(0.2247)
*CS*	0.1359 *	−0.0367	−0.2518 ***	0.1181 *	−0.0377	−0.2677 ***
	(0.0704)	(0.0748)	(0.0748)	(0.0716)	(0.0746)	(0.0739)
*VLD*	−0.0169	−0.0570	−0.1576 **	0.0149	−0.0558	−0.1440 **
	(0.0542)	(0.0553)	(0.0618)	(0.0558)	(0.0569)	(0.0624)
*GEN*	−0.1679	−0.1095	−0.0491	−0.1811	−0.1135	−0.0538
	(0.1557)	(0.1549)	(0.1656)	(0.1573)	(0.1547)	(0.1670)
*AGE*	0.0598	0.3379 ***	0.1583	0.0204	0.3346 ***	0.1336
	(0.1031)	(0.1069)	(0.1046)	(0.1030)	(0.1066)	(0.1047)
*EDU*	0.0615	0.3618 ***	0.3349 ***	−0.0578	0.3536 ***	0.2748 ***
	(0.0926)	(0.0918)	(0.0938)	(0.0990)	(0.0957)	(0.1020)
*HI*	0.1684 *	−0.0207	0.1269	0.1503 *	−0.0225	0.1135
	(0.0871)	(0.0820)	(0.0970)	(0.0894)	(0.0826)	(0.0964)
*CLS*	−0.0411	−0.0148	0.0651 *	−0.0387	−0.0150	0.0694 *
	(0.0371)	(0.0366)	(0.0391)	(0.0379)	(0.0365)	(0.0390)
*HPS*	0.9863 ***	0.9004 ***	0.5966	0.9551 ***	0.8985 ***	0.5519
	(0.3454)	(0.3368)	(0.3802)	(0.3533)	(0.3375)	(0.3810)
Constant	−1.8246 ***	−1.4432 **	−0.9057	−1.5018 **	−1.4073 **	−0.6793
	(0.6448)	(0.6464)	(0.6927)	(0.6432)	(0.6498)	(0.6944)
atrho21		0.4177 ***			0.4323 ***	
		(0.1043)			(0.1066)	
atrho31		0.1530			0.1233	
		(0.1031)			(0.1039)	
atrho32		0.4634 ***			0.4663 ***	
		(0.1050)			(0.1053)	
Obs		306			306	
Draws		18			18	
Wald Value		107.21 ***			117.81 ***	

Source: compiled by the author. Notes: Robust standard errors in parentheses, ***, **, and * indicate significance at the 1%, 5% and 10% levels, respectively.

**Table 4:** MVP model robustness test results.

Variable	(1)	(2)	(3)	(4)	(5)	(6)
	*Y*_1_	*Y*_2_	*Y*_3_	*Y*_1_	*Y*_2_	*Y*_3_
*SIDJ*	0.0272	−0.1616 **	−0.1737 **	0.0252	−0.1644 **	−0.1763 **
	(0.0713)	(0.0693)	(0.0751)	(0.0716)	(0.0691)	(0.0755)
*GB*				0.6602 ***	0.0376	0.3365
				(0.2176)	(0.2154)	(0.2248)
*CS*	0.1245 *	−0.0388	−0.2497 ***	0.1060	−0.0397	−0.2654 ***
	(0.0708)	(0.0748)	(0.0747)	(0.0719)	(0.0745)	(0.0739)
*VLD*	−0.0202	−0.0734	−0.1731 ***	0.0109	−0.0723	−0.1597 **
	(0.0537)	(0.0554)	(0.0617)	(0.0554)	(0.0570)	(0.0624)
*GEN*	−0.1585	−0.1037	−0.0498	−0.1702	−0.1081	−0.0545
	(0.1558)	(0.1546)	(0.1655)	(0.1575)	(0.1544)	(0.1669)
*AGE*	0.0671	0.3340 ***	0.1498	0.0281	0.3306 ***	0.1255
	(0.1036)	(0.1070)	(0.1043)	(0.1034)	(0.1067)	(0.1049)
*EDU*	0.0715	0.3549 ***	0.3235 ***	−0.0466	0.3469 ***	0.2630 **
	(0.0927)	(0.0924)	(0.0949)	(0.0991)	(0.0961)	(0.1035)
*HI*	0.1775 **	0.0133	0.1576 *	0.1600 *	0.0117	0.1447
	(0.0845)	(0.0811)	(0.0946)	(0.0867)	(0.0818)	(0.0943)
*CLS*	−0.0428	−0.0168	0.0635	−0.0410	−0.0172	0.0678 *
	(0.0370)	(0.0365)	(0.0391)	(0.0380)	(0.0364)	(0.0391)
*HPS*	0.9827 ***	0.8535 **	0.5545	0.9501 ***	0.8510 **	0.5104
	(0.3466)	(0.3358)	(0.3796)	(0.3541)	(0.3365)	(0.3803)
Constant	−1.9671 ***	−1.4331 **	−0.8162	−1.6487 **	−1.3950 **	−0.5911
	(0.6512)	(0.6488)	(0.6980)	(0.6470)	(0.6522)	(0.7065)
atrho21		0.4261 ***			0.4416 ***	
		(0.1049)			(0.1073)	
atrho31		0.1658			0.1374	
		(0.1035)			(0.1042)	
atrho32		0.4651 ***			0.4682 ***	
		(0.1049)			(0.1054)	
Obs		306			306	
Draws		18			18	
Wald Value		106.01 ***			116.61 ***	

Source: compiled by the author. Notes: Robust standard errors in parentheses, ***, **, and * indicate significance at the 1%, 5% and 10% levels, respectively.

## Data Availability

Not applicable.
